# Supplementary material for: Reduced expression of C/EBPβ-LIP extends health and lifespan in mice
Source: eLife. 2018 Jun 4;7:e34985. doi: 10.7554/eLife.34985 (PMC5986274; doi:10.7554/eLife.34985)
Supplement: Supplementary file 1. — 1 Number of animals in the cohort 2 Median survival of the cohort (days) 3 Increase of the median survival (percent) 4 P-value of the increased survival (log-rank test) 5 Mean lifespan of the cohort (days) 6 Standard error of the mean 7 Number of mice in the cohort in the longest-lived decile of the combined cohort (wt and C/EBPβΔuORF) 8 P-value of increased Nmax7 (Fisher’s exact test). [file elife-34985-supp1.docx]

**Supplementary file 1 - Table 1**

**Lifespan experiment summary of results**

| **Genotype** | **sex** | **N^1^** | **Median^2^** | **Increase^3^** | **P_Surv_^4^** | **Mean^5^** | **SEM^6^** | **N_max_^7^** | **P_max_^8^** |
| --- | --- | --- | --- | --- | --- | --- | --- | --- | --- |

| **All mice** | | | | | | | | | |
| --- | --- | --- | --- | --- | --- | --- | --- | --- | --- |
| C/EBPβ^ΔuORF^ | Females | 50 | 751.5 | 20.63 | 0.0014 | 747.42 | 23.7 | 9 | 0.0157 |
| wt | Females | 50 | 623.0 |  |  | 650.96 | 22.5 | 1 |  |
| C/EBPβ^ΔuORF^ | Males | 50 | 833,5 | 5.24 | 0.4647 | 781.90 | 28.8 | 6 | 0.7407 |
| wt | Males | 50 | 792.0 |  |  | 777.68 | 24.8 | 4 |  |
| C/EBPβ^ΔuORF^ | Both | 100 | 797.0 | 10.54 | 0.0323 | 764.66 | 18.6 | 13 | 0.2381 |
| wt | Both | 100 | 721.0 |  |  | 714.32 | 17.8 | 7 |  |

| **Mice ulcerative dermatitis (UD)-free** | | | | | | | | | |
| --- | --- | --- | --- | --- | --- | --- | --- | --- | --- |
| C/EBPβ^ΔuORF^ | Females | 24 | 860.5 | 16.28 | 0.0017 | 809.29 | 34.10 | 5 | 0.0755 |
| wt | Females | 31 | 740.0 |  |  | 686.07 | 29.71 | 1 |  |
| C/EBPβ^ΔuORF^ | Males | 40 | 857.5 | 3.44 | 0.8282 | 789.10 | 33.11 | 6 | 0.2710 |
| wt | Males | 35 | 829.0 |  |  | 821.49 | 24.75 | 2 |  |
| C/EBPβ^ΔuORF^ | Both | 64 | 857.5 | 10.86 | 0.0804 | 796.67 | 24.19 | 8 | 0.3936 |
| wt | Both | 66 | 773.5 |  |  | 757.88 | 20.78 | 5 |  |

| **Mice with UD** | | | | | | | | | |
| --- | --- | --- | --- | --- | --- | --- | --- | --- | --- |
| C/EBPβ^ΔuORF^ | Females | 26 | 711.5 | 22.88 | 0.0122 | 690.31 | 37.37 | 5 | 0.0634 |
| wt | Females | 19 | 579.0 |  |  | 593.68 | 30.65 | 0 |  |
| C/EBPβ^ΔuORF^ | Males | 10 | 802.5 | 12.40 | 0.3299 | 753.1 | 58.63 | 1 | 1.0 |
| wt | Males | 15 | 714.0 |  |  | 675.47 | 51.28 | 2 |  |
| C/EBPβ^ΔuORF^ | Both | 36 | 733.5 | 14.88 | 0.0488 | 707.75 | 26.67 | 5 | 0.4297 |
| wt | Both | 34 | 638.5 |  |  | 629.77 | 28.78 | 2 |  |
