# Supplementary material for: Reduced expression of C/EBPβ-LIP extends health and lifespan in mice
Source: eLife. 2018 Jun 4;7:e34985. doi: 10.7554/eLife.34985 (PMC5986274; doi:10.7554/eLife.34985)
Supplement: Supplementary file 2. — *incl. lymphoid leukaemia **incl. malignant round cell neoplasms ***tumour type could not be unequivocally determined due to inadequate quality of the fixed tumour tissue Absolute numbers of mice with the indicated tumour type of tumours found during necropsy for wt and C/EBPβΔuORF males and females are shown. Note that the total number of tumours is higher than the number of tumour-bearing mice due to the eventual occurrence of different tumour types in the same mouse. [file elife-34985-supp2.docx]

**Supplementary file 2 - Table 2**

**Tumour spectrum in wt and C/EBPβ^ΔuORF^ mice**

| Tumour type | Location | wt  females | ΔuORF  females | wt  males | ΔuORF males |
| --- | --- | --- | --- | --- | --- |
| Lymphoma * | different | 15 | 6 | 1 | 4 |
| Hepatocellular carcinoma | liver | 8 | 4 | 14 | 6 |
| Histiocytic sarcoma | liver, colon, intestine, spleen | 8 | 3 | 14 | 9 |
| Myeloid neoplasms ** | different | 4 | 4 | 4 | 6 |
| Adenocarcinoma | pituitary, mammary gland, haderian gland, intestine | 2 | 2 | 0 | 3 |
| Bronchio-alveolar carcinoma | lung | 0 | 1 | 0 | 3 |
| Mast cell tumour | stomach, liver | 1 | 0 | 0 | 1 |
| Anaplastic sarcoma | leg, side | 1 | 1 | 0 | 0 |
| Osteosarcoma | leg | 1 | 0 | 0 | 0 |
| Undifferentiated sarcoma | pancreas, colon, kidney | 0 | 0 | 0 | 1 |
| Squamous cell carcinoma | penis | 0 | 0 | 1 | 0 |
| Benign neoplasms | bladder, neck, spleen, liver | 3 | 1 | 1 | 0 |
| Unidentified*** | liver, kidney, colon, spleen, lung, throat, abdominal cavity, adrenal gland | 6 | 5 | 3 | 2 |
