# Supplementary material for: Reduced expression of C/EBPβ-LIP extends health and lifespan in mice
Source: eLife. 2018 Jun 4;7:e34985. doi: 10.7554/eLife.34985 (PMC5986274; doi:10.7554/eLife.34985)
Supplement: Supplementary file 3. — Mice were part of the ageing cohort and the age at analysis was 20 months for females and 22 months for males. 1 Number of animals showing the pathology (out of the total number of animals analyzed). 2 mean grade of the pathology as calculated from the total number of animals analyzed with 0 = absent, 1 = mild, 2 = moderate and 3 = severe. 3 Statistical significance of difference found between wt and C/EBPβΔuORF mice from the same gender as calculated using the Student’s t-test (ns = not significant). 4 Mean number of regenerating muscle fibers found in five histological tissue slices per mouse. Note that a lower number is an indication for a more progressed ageing phenotype. 5 mean surface area of intramuscular adipose tissue in percent of the total area of analyzed skeletal muscle tissue as calculated from the total number of animals analyzed. 6 Trabecular bone parameters (percent bone volume/tissue volume; Trabecular number per mm; trabecular thickness and trabecular separation) measured by micro-CT analysis. [file elife-34985-supp3.docx]

**Supplementary file 3 -Table 3**

**Occurrence of ageing-associated pathologies in wt and C/EBPβ^ΔuORF^ mice**

| Tissue | Pathology/Histology |  | wt females | ΔuORF females | wt males | ΔuORF males |
| --- | --- | --- | --- | --- | --- | --- |
| Liver | Hepatocellular vacuolation | Number^1^  Grade^2^ ± SEM  Significance^3^ | 9 (9)  1.06 ± 0.19 | 9 (9)  1.06 ± 0.13 | 9 (9)  1.5 ± 0.20 | 9 (9)  0.67 ± 0.12  p < 0.01 |
|  | Cytoplasmic nuclear inclusions | Number^1^  Grade^2^ ± SEM  Significance^3^ | 2 (9)  0.06 ± 0.04 | 2 (9)  0.14 ± 0.11  ns | 9 (9)  1.11 ± 0.14 | 5 (9)  0.33 ± 0.14  p < 0.01 |
|  | Polyploidy | Number^1^  Grade^2^ ± SEM  Significance^3^ | 9 (9)  1.39 ± 0.11 | 9 (9)  1,72 ± 0.17 ns | 9 (9)  2.28 ± 0.09 | 9 (9)  2.06 ± 0.10 ns |
|  | Lymphoplasmatic  inflammation | Number^1^  Grade^2^ ± SEM  Significance^3^ | 4 (9)  0.44 ± 0.19 | 8 (9)  1.28 ± 0.28  p < 0.05 | 4 (9)  0.39 ± 0.22 | 7 (9)  0.72 ± 0.21  ns |
| Pancreas | Islet cell hyperplasia | Number^1^  Grade^2^ ± SEM  Significance^3^ | 7 (9)  0.72 ± 0.17 | 4 (9)  0.28 ± 0.12  p < 0.05 | 6 (9)  0.50 ± 0.17 | 1 (9)  0.06 ± 0.06  p < 0.05 |
|  | Focal acinar cell atrophy | Number^1^  Grade^2^ ± SEM  Significance^3^ | 0 (9)  0 | 2 (9)  0.11 ± 0.07 ns | 2 (9)  0.17 ± 0.12 | 1 (9)  0.06 ± 0.06 ns |
|  | Fatty replacement of acinar cells | Number^1^  Grade^2^ ± SEM  Significance^3^ | 0 (9)  0 | 2 (9)  0.17 ± 0.12 ns | 2 (9)  0.17 ± 0.12 | 1 (9)  0.06 ± 0.06 ns |
|  | Inflammation | Number^1^  Grade^2^ ± SEM  Significance^3^ | 6 (9)  0.39 ± 0.11 | 7 (9)  0.61 ± 0.16 ns | 3 (9)  0.17 ± 0.08 | 6 (9)  0.33 ± 0.08 ns |
| Skeletal muscle | Regenerative muscle fibers | Number^1^  No.^4^ ± SEM  Significance^3^ | 1 (8)  0.13 ± 0.13 | 3 (9)  0.78 ± 0.55 ns | 3 (9)  0.78 ± 0.40 | 6 (9)  6.33 ± 2.13  p < 0.05 |
|  | Nuclear condensation | Number^1^  Grade^2^ ± SEM  Significance^3^ | 3 (9)  0.22 ± 0.12 | 7 (9)  0.78 ± 0.22  p < 0.05 | 9 (9)  1.22 ± 0.21 | 7 (9)  1.22 ± 0.28 |
|  | Intramuscular adipose tissue | Number^1^  Grade^2^ ± SEM  Significance^3^ | 6 (9)  2.89 ± 1.11 | 7 (9)  2.24 ± 1.15 ns | 5 (9)  2.82 ± 2.93 | 6 (9)  8.33 ± 2.92 ns |
| Spleen | Lymphoid hyperplasia | Number^1^  Grade^2^ ± SEM  Significance^3^ | 8 (9)  1.83 ± 0.26 | 9 (9)  1.72 ± 0.12 ns | 9 (9)  1.67 ± 0.14 | 9 (9)  1.72 ± 0.17 ns |
|  | Extramedullary hematopoiesis | Number^1^  Grade^2^ ± SEM  Significance^3^ | 9 (9)  1.50 ± 0.24 | 9 (9)  1.06 ± 0.16  ns | 9 (9)  1.11 ± 0.14 | 9 (9)  1.28 ± 0.22 ns |
|  | Dendritic reticular cell hyperplasia | Number^1^  Grade^2^ ± SEM  Significance^3^ | 4 (9)  0.28 ± 0.12 | 2 (9)  0.22 ± 0.15 ns | 4 (9)  0.22 ± 0.09 | 4 (9)  0.56 ± 0.28 ns |
| Skin | Dermal inflammation | Number^1^  Grade^2^ ± SEM  Significance^3^ | 9 (9)  0.89 ± 0.16 | 8 (9)  0.44 ± 0.06  p < 0.05 | 9 (9)  0.39 ± 0.04 | 9 (9)  0.39 ± 0.04 |
| Bone | Bone volume^6^ | Bone vol. / tissue vol. (%) ± SEM | 0.56 ± 0.11  (n=5) | 0.51 ± 0.27  ns (n=5) | 3.94 ± 0.42  (n=5) | 3.69 ±1.14  ns (n=5) |
|  | Trabecular number^6^ | Number (1/μm) ± SEM | 9.00x10^-5^ ± 1.48x10^-5^  (n=5) | 9,40x10^-5^ ± 5.14x10^-5^  ns (n=5) | 4.94 x10^-4^ ± 5.86x10^-5^  (n=5) | 5,66x10^-4^ ± 1.75x10^-4^  ns (n=5) |
|  | Trabecular thickness^6^ | Thickness (μm) ± SEM | 62.27 ± 3.97  (n=5) | 54,63 ± 10,35  ns (n=5) | 80.94 ± 4.58  (n=5) | 65.50 ± 1.64  p < 0.05 (n=5) |
|  | Trabecular separation^6^ | Distance (μm) ± SEM | 697,55 ± 48.59  (n=5) | 777.81 ± 50.82  ns (n=5) | 469.05 ± 36.98  (n=5) | 489.07 ± 42.27  ns (n=5) |
| Blood | IGF-1 levels (plasma)^9^ | ng/ml ± SEM  Significance^3^ | 375.4 ± 17.8  (n=11) | 321.5 ± 16.4  p < 0.05  (n=11) | 291.2 ± 21.9  (n=12) | 238.6 ± 22.5  ns (n =10) |
