# Supplementary material for: Reduced expression of C/EBPβ-LIP extends health and lifespan in mice
Source: eLife. 2018 Jun 4;7:e34985. doi: 10.7554/eLife.34985 (PMC5986274; doi:10.7554/eLife.34985)
Supplement: Supplementary file 4. — Functional annotation of genes upregulated in livers of old C/EBPβΔuORF female mice compared to livers of old wt female mice (FDR < 0.01; 103 from 127 genes; 24 unknown IDs) using the DAVID database (Huang et al., 2009). [file elife-34985-supp4.docx]

**Supplementary file 4 - Table 4**

**GO-term analysis of genes upregulated in livers of old C/EBPβ^ΔuORF^ mice**

| **GO term** | **Description** | **p-value** | **FDR** | **Number of genes** | **Fold enrich-ment** |
| --- | --- | --- | --- | --- | --- |
| GO:0009897 | External side of plasma membrane | 4.4x10^-11^ | 5.2x10^-8^ | 16 | 10.1 |
| GO:0042102 | Positive regulation of T cell proliferation | 2.5x10^-8^ | 3.8x10^-5^ | 8 | 25.4 |
| GO:0016020 | Membrane | 3.9x10^-8^ | 4.7x10^-5^ | 62 | 1.8 |
| GO:0031295 | T cell costimulation | 1.2x10^-7^ | 1.8x10^-4^ | 6 | 48.8 |
| GO:0001772 | Immunological synapse | 6.5x10^-7^ | 7.8x10^-4^ | 6 | 35.4 |
| GO:0006955 | Immune response | 8.1x10^-7^ | 1.2x10^-3^ | 11 | 8.2 |
| GO:0042113 | B cell activation | 1.1x10^-5^ | 1.7x10^-2^ | 5 | 35.0 |
| GO:0046641 | Positive regulation of alpha-beta T cell proliferation | 1.8x10^-5^ | 2.7x10^-2^ | 4 | 73,9 |
| GO:0045086 | Positive regulation of interleukine-2 biosynthetic process | 2.4x10^-5^ | 3.5x10^-2^ | 4 | 67.7 |

Functional annotation of genes upregulated in livers of old C/EBPβ^ΔuORF^ female mice compared to livers of old wt female mice (FDR < 0.01; 103 from 127 genes; 24 unknown IDs) using the DAVID database (Huang et al., 2009)
