# Supplementary material for: Reduced expression of C/EBPβ-LIP extends health and lifespan in mice
Source: eLife. 2018 Jun 4;7:e34985. doi: 10.7554/eLife.34985 (PMC5986274; doi:10.7554/eLife.34985)
Supplement: Supplementary file 5. — Functional annotation of genes downregulated in livers of old C/EBPβΔuORF female mice compared to livers of old wt female mice (FDR < 0.01; 23 from 25 genes, two unknown IDs) using the DAVID database (Huang et al., 2009). [file elife-34985-supp5.docx]

**Supplementary file 5 - Table 5**

**GO-term analysis of genes downregulated in livers of old C/EBPβ^ΔuORF^ mice**

| **GO term** | **Description** | **p-value** | **FDR q-value** | **Number of genes** | **Fold enrich-ment** |
| --- | --- | --- | --- | --- | --- |
| GO:0006953 | Acute phase response | 5.3x10^-6^ | 6.2x10^-3^ | 4 | 108.8 |
| GO:0005576 | Extracellular space | 2.5x10^-5^ | 2.4x10^-2^ | 9 | 6.2 |
