# Supplementary material for: Reduced expression of C/EBPβ-LIP extends health and lifespan in mice
Source: eLife. 2018 Jun 4;7:e34985. doi: 10.7554/eLife.34985 (PMC5986274; doi:10.7554/eLife.34985)
Supplement: Supplementary file 6. — Functional annotation of genes showing a high inter-individual variation between livers from old wt female mice. Coefficient of variation of transcript levels of the corresponding gene in the livers of wt mice is at least twice as big as the coefficient of variation of the transcript levels of the same gene in the livers from C/EBPβΔuORF mice; 1386 from 1414 genes, 28 unknown IDs; using the DAVID database (Huang et al., 2009). [file elife-34985-supp6.docx]

**Supplementary file 6 - Table 6**

**GO-term analysis of genes showing high inter-individual variation between livers of old wt female mice**

| **GO term** | **Description** | **p-value** | **FDR q-value** | **Number of genes** | **Fold enrich-ment** |
| --- | --- | --- | --- | --- | --- |
| GO:0005739 | Mitochondrion | 1.2x10^-30^ | 1.8x10^-27^ | 236 | 2.1 |
| GO:0070062 | Extracellular exosome | 3.4x10^-29^ | 5.1x10^-26^ | 315 | 1.8 |
| GO:0055114 | Oxidation-reduction process | 4.4x10^-26^ | 8.0x10^-23^ | 124 | 2.8 |
| GO:0005743 | Mitochondrial inner membrane | 2.5x10^-24^ | 3.7x10^-21^ | 86 | 3.5 |
| GO:0016491 | Oxidoreductase activity | 2.6x10^-24^ | 4.2x10^-21^ | 113 | 2.8 |
| GO:0005783 | Endoplasmic reticulum | 1.8x10^-17^ | 2.6x10^-14^ | 166 | 2.0 |
| GO:0008152 | Metabolic process | 1.7x10^-12^ | 3.1x10^-9^ | 74 | 2.4 |
| GO:0072562 | Blood microparticle | 5.0x10^-11^ | 7.3x10^-8^ | 33 | 3.9 |
| GO:0006953 | Acute phase response | 1.2x10^-10^ | 2.3x10^-7^ | 17 | 7.4 |
| GO:0005789 | Endoplasmic reticulum membrane | 1.6x10^-10^ | 2.4x10^-7^ | 92 | 2.0 |
| GO:0006810 | Transport | 2.3x10^-10^ | 4.2x10^-7^ | 187 | 1.6 |
| GO:0043231 | Intracellular membrane-bounded organelle | 2.9x10^-10^ | 4.3x10^-7^ | 95 | 2.0 |
| GO:0007596 | Blood coagulation | 2.5x10^-9^ | 4.5x10^-6^ | 24 | 4.4 |
| GO:0042730 | Fibrinolysis | 3.0x10^-9^ | 5.6x10^-6^ | 11 | 11.2 |
| GO:0016020 | Membrane | 4.5x10^-9^ | 6.6x10^-6^ | 546 | 1.2 |
| GO:0006635 | Fatty acid beta-oxidation | 5.9x10^-8^ | 1.1x10^-4^ | 16 | 5.6 |
| GO:0006631 | Fatty acid metabolic process | 8.0x10^-8^ | 1.5x10^-4^ | 31 | 3.0 |
| GO:0031090 | Organelle membrane | 8.7x10^-8^ | 1.3x10^-4^ | 23 | 3.8 |
| GO:0006629 | Lipid metabolic process | 9.2x10^-8^ | 1.7x10^-4^ | 62 | 2.1 |
| GO:0007599 | Hemostasis | 1.2x10^-7^ | 2.1x10^-4^ | 16 | 5.3 |
| GO:0005777 | Peroxisome | 2.1x10^-7^ | 3.1x10^-4^ | 27 | 3.2 |
| GO:0006749 | Glutathione metabolic process | 3.0x10^-7^ | 5.5x10^-4^ | 16 | 5.0 |
| GO:0005829 | Cytosol | 4.6x10^-7^ | 6.7x10^-4^ | 167 | 1.5 |
| GO:0005615 | Extracellular space | 9.5x10^-7^ | 1.4x10^-3^ | 144 | 1.5 |
| GO:0034364 | High-density lipoprotein particle | 2.6x10^-6^ | 3.8x10^-3^ | 10 | 7.4 |
| GO:0005793 | Endoplasmic reticulum-Golgi intermediate compartment | 4.6x10^-6^ | 6.7x10^-3^ | 17 | 3.9 |
| GO:0004364 | Glutathione transferase activity | 4.8x10^-6^ | 7.8x10^-3^ | 12 | 5.5 |
| GO:0004129 | Cytochrome-c oxidase activity | 6.4x10^-6^ | 1.1x10^-2^ | 11 | 6.0 |
| GO:0003988 | Acetyl-CoA C-acetyltransferase activity | 6.9x10^-6^ | 1.1x10^-2^ | 6 | 15.2 |
| GO:0006099 | Tricarboxylic acid cycle | 8.7x10^-6^ | 1.6x10^-2^ | 11 | 5.8 |
| GO:0020037 | Heme binding | 1.1x10^-5^ | 1.7x10^-2^ | 29 | 2.5 |
| GO:0043022 | Ribosome binding | 1.1x10^-5^ | 1.8x10^-2^ | 15 | 4.1 |
| GO:0016829 | Lyase activity | 1.2x10^-5^ | 2.0x10^-2^ | 25 | 2.7 |
| GO:0005765 | Lysosomal membrane | 1.7x10^-5^ | 2.5x10^-2^ | 34 | 2.3 |
| GO:0016787 | Hydrolase activity | 2.2x10^-5^ | 3.6x10^-2^ | 142 | 1.4 |
| GO:0004497 | Monooxygenase activity | 2.2x10^-5^ | 3.6x10^-2^ | 21 | 3.0 |
