# Supplementary material for: Reduced expression of C/EBPβ-LIP extends health and lifespan in mice
Source: eLife. 2018 Jun 4;7:e34985. doi: 10.7554/eLife.34985 (PMC5986274; doi:10.7554/eLife.34985)
Supplement: Supplementary file 7. — Functional annotation of genes showing a high inter-individual variation between livers from old wt female mice. Coefficient of variation of transcript levels of the corresponding gene in the livers of C/EBPβΔuORF mice is at least twice as big as the coefficient of variation of the transcript levels of the same gene in the livers from wt mice; 1354 from 1375 genes, 21 unknown IDs; using the DAVID database (Huang et al., 2009). [file elife-34985-supp7.docx]

**Supplementary file 7 - Table 7**

**GO-term analysis of genes showing high inter-individual variation between livers of old C/EBPβ^ΔuORF^ female mice**

| **GO term** | **Description** | **p-value** | **FDR q-value** | **Number of genes** | **Fold enrich-ment** |
| --- | --- | --- | --- | --- | --- |
| GO:0005634 | Nucleus | 8.9x10^-45^ | 1.3x10^-41^ | 596 | 1.6 |
| GO:0005654 | Nucleoplasm | 7.1x10^-35^ | 1.1x10^-31^ | 258 | 2.2 |
| GO:0007049 | Cell cycle | 1.1x10^-26^ | 2.0x10^-23^ | 116 | 3.0 |
| GO:0005737 | Cytoplasm | 1.4x10^-25^ | 2.1x10^-21^ | 577 | 1.4 |
| GO:0003723 | Poly(A) RNA binding | 1.8x10^-20^ | 2.8x10^-17^ | 155 | 2.2 |
| GO:0005515 | Protein binding | 2.4x10^-16^ | 3.6x10^-13^ | 381 | 1.4 |
| GO:0006260 | DNA replication | 6.1x10^-17^ | 1.6x10^-13^ | 37 | 6.1 |
| GO:0000166 | Nucleotide binding | 1.2x10^-14^ | 1.8x10^-11^ | 210 | 1.7 |
| GO:0051301 | Cell division | 2.6x10^-13^ | 4.8x10^-10^ | 65 | 2.7 |
| GO:0005524 | ATP binding | 2.6x10^-13^ | 4.2x10^-10^ | 170 | 1.7 |
| GO:0006351 | Transcription, DNA-templated | 1.6x10^-11^ | 2.9x10^-8^ | 192 | 1.6 |
| GO:0003677 | DNA binding | 3.6x10^-11^ | 5.8x10^-8^ | 190 | 1.6 |
| GO:0000278 | Mitotic nuclear division | 5.1x10^-11^ | 9.3x10^-8^ | 50 | 2.8 |
| GO:0005730 | Nucleolus | 6.0x10^-11^ | 9.1x10^-8^ | 102 | 2.0 |
| GO:0006974 | Cellular response to DNA damage stimulus | 1.4x10^-10^ | 2.5x10^-7^ | 64 | 2.4 |
| GO:0003682 | Chromatin binding | 1.2x10^-9^ | 1.9x10^-6^ | 67 | 2.2 |
| GO:0003723 | RNA binding | 5.1x10^-9^ | 8.1x10^-6^ | 94 | 1.9 |
| GO:0006281 | DNA repair | 6.4x10^-9^ | 1.2x10^-5^ | 50 | 2.5 |
| GO:0004386 | Helicase activity | 1.0x10^-8^ | 1.7x10^-5^ | 29 | 3.5 |
| GO:0006355 | Regulation of transcription,  DNA templated | 2.7x10^-8^ | 4.9x10^-5^ | 208 | 1.4 |
| GO:0002376 | Immune system process | 1.6x10^-7^ | 3.0x10^-4^ | 53 | 2.2 |
| GO:0005643 | Nuclear pore | 2.0x10^-7^ | 3.0x10^-4^ | 18 | 4.6 |
| GO:0005694 | Chromosome | 2.2x10^-7^ | 3.3x10^-4^ | 48 | 2.3 |
| GO:0043234 | Protein complex | 2.3x10^-7^ | 3.4x10^-4^ | 73 | 1.9 |
| GO:0045893 | Positive regulation of transcription,  DNA templated | 2.4x10^-7^ | 4.4x10^-4^ | 70 | 1.9 |
| GO:0006397 | mRNA processing | 4.8x10^-7^ | 8.9x10^-4^ | 46 | 2.3 |
| GO:0009615 | Response to virus | 9.5x10^-7^ | 1.7x10^-3^ | 20 | 3.8 |
| GO:0042393 | Histone binding | 1.1x10^-6^ | 1.7x10^-3^ | 25 | 3.1 |
| GO:0004402 | Histone acetyltransferase activity | 1.4x10^-6^ | 2.2x10^-3^ | 14 | 5.2 |
| GO:0008380 | RNA splicing | 1.4x10^-6^ | 2.5x10^-3^ | 37 | 2.4 |
| GO:0003725 | Double-stranded RNA binding | 1.5x10^-6^ | 2.5x10^-3^ | 18 | 4.0 |
| GO:0005681 | Spliceosomal complex | 2.6x10^-6^ | 3.9x10^-3^ | 25 | 3.0 |
| GO:0008283 | Cell proliferation | 3.4x10^-6^ | 6.3x10^-3^ | 34 | 2.4 |
| GO:0042555 | MCM complex | 3.8x10^-6^ | 5.7x10^-3^ | 7 | 12.6 |
| GO:0005856 | Cytoskeleton | 3.9x10^-6^ | 5.9x10^-3^ | 107 | 1.6 |
| GO:0016569 | Covalent chromatin modification | 5.5x10^-6^ | 1.0x10^-2^ | 38 | 2.3 |
| GO:0030529 | Intracellular ribonucleoprotein complex | 6.6x10^-6^ | 1.0x10^-2^ | 42 | 2.1 |
| GO:0000122 | Negative regulation of transcription from RNA polymerase II promoter | 6.7x10^-6^ | 1.2x10^-2^ | 78 | 1.7 |
| GO:0051028 | mRNA transport | 8.3x10^-6^ | 1.5x10^-2^ | 19 | 3.4 |
| GO:0000281 | Mitotic cytokinesis | 9.4x10^-6^ | 1.7x10^-2^ | 11 | 5.8 |
| GO:0005813 | Centrosome | 1.5x10^-5^ | 2.2x10^-2^ | 51 | 1.9 |
| GO:0000775 | Chromosome, centromeric region | 1.6x10^-5^ | 2.4x10^-2^ | 24 | 2.8 |
| GO:0005829 | Cytosol | 1.8x10^-5^ | 2.7x10^-2^ | 153 | 1.4 |
| GO:0043967 | Histone H4 acetylation | 1.8x10^-5^ | 3.3x10^-2^ | 11 | 5.4 |
| GO:0003697 | Single-stranded DNA binding | 1.8x10^-5^ | 2.9x10^-2^ | 18 | 3.4 |
| GO:0016607 | Nuclear speck | 2.3x10^-5^ | 3.5x10^-2^ | 30 | 2.4 |
| GO:0005874 | Microtubule | 2.5x10^-5^ | 3.8x10^-2^ | 41 | 2.0 |
| GO:0000784 | Nuclear chromosome, telomeric region | 3.0x10^-5^ | 4.5x10^-2^ | 22 | 2.8 |
