# Supplementary material for: Reduced expression of C/EBPβ-LIP extends health and lifespan in mice
Source: eLife. 2018 Jun 4;7:e34985. doi: 10.7554/eLife.34985 (PMC5986274; doi:10.7554/eLife.34985)
Supplement: Transparent reporting form [file elife-34985-transrepform.docx]

***eLife’s* transparent reporting form**

We encourage authors to provide detailed information *within their submission* to facilitate the interpretation and replication of experiments. Authors can upload supporting documentation to indicate the use of appropriate reporting guidelines for health-related research (see [EQUATOR Network](http://www.equator-network.org/%20)), life science research (see the [BioSharing Information Resource](https://biosharing.org/)), or the [ARRIVE guidelines](http://www.plosbiology.org/article/info:doi/10.1371/journal.pbio.1000412) for reporting work involving animal research. Where applicable, authors should refer to any relevant reporting standards documents in this form.

If you have any questions, please consult our Journal Policies and/or contact us: [editorial@elifesciences.org](mailto:editorial@elifesciences.org).

**Sample-size estimation**

- You should state whether an appropriate sample size was computed when the study was being designed
- You should state the statistical method of sample size computation and any required assumptions
- If no explicit power analysis was used, you should describe how you decided what sample (replicate) size (number) to use

Please outline where this information can be found within the submission (e.g., sections or figure legends), or explain why this information doesn’t apply to your submission:

- The Sample sizes for quantification of the Western blots shown in Figure 1B,C and of the related Figure 1-supplement 1 are mentioned in the figure legends.

- The Sample sizes Figure 1D,E are mentioned in the figure legend.

- The Sample sizes for the Figures 2A,B and Figure 2-supplement 1A, D, E are mentioned in the main text, in the Material and Methods section, within the figures and in the Supplementary file 1 (Table 1).

- The sample sizes for the Figures 2C, D, E and 2F and Figure 2-supplements 1B and C are mentioned within the figures and in the Supplementary file 1 (Table 1).

- The sample sizes for Figures 3A,B are mentioned in the figure legend.

- The sample sizes for Figure supplement 3A,C,E,F are mentioned within the Figure.

- The sample sizes for Figure 4A,B,C,D,E,F and for Figure 2-supplement 1A,B,C,D are found in the figure legends.

- The sample sizes for Figure 5A,B,C,D and Figure 5-supplement 1A,B,C,D are found in the figure legends.

- The sample sizes for Figure 6A,B,C,D are found in the figure legend.

- The sample size for Figure 7-supplement 1 is mentioned in the figure legend.

- The sample sizes for Supplemental files 1, and 3 are given in the tables.

- The sample sizes for Supplemental files 4, 5, 6 and 7 are given in the table legends.

**Replicates**

- You should report how often each experiment was performed
- You should include a definition of biological versus technical replication
- The data obtained should be provided and sufficient information should be provided to indicate the number of independent biological and/or technical replicates
- If you encountered any outliers, you should describe how these were handled
- Criteria for exclusion/inclusion of data should be clearly stated
- High-throughput sequence data should be uploaded before submission, with a private link for reviewers provided (these are available from both GEO and ArrayExpress)

Please outline where this information can be found within the submission (e.g., sections or figure legends), or explain why this information doesn’t apply to your submission:

The reproducibility of the experiments was secured by performing biological replicates. The number of replicates is mentioned (n=x) in the figure legends. The information about the kind of replicates is mentioned in the Statistical analysis paragraph of the Material and methods section.

The information about exclusion of outliers is found in the Material and methods section.

The Results and Material and methods section contain a citation for the RNA-seq dataset in the reference list, which contains data base name (EBI ArrayExpress) and URL.

**Statistical reporting**

- Statistical analysis methods should be described and justified
- Raw data should be presented in figures whenever informative to do so (typically when N per group is less than 10)
- For each experiment, you should identify the statistical tests used, exact values of N, definitions of center, methods of multiple test correction, and dispersion and precision measures (e.g., mean, median, SD, SEM, confidence intervals; and, for the major substantive results, a measure of effect size (e.g., Pearson's r, Cohen's d)
- Report exact p-values wherever possible alongside the summary statistics and 95% confidence intervals. These should be reported for all key questions and not only when the p-value is less than 0.05.

Please outline where this information can be found within the submission (e.g., sections or figure legends), or explain why this information doesn’t apply to your submission:

The general information about the statistical analysis methods used is mentioned in the Statistical analysis paragraph of the Material and methods section.

The information about the statistical tests and dispersion method used for each experiment is mentioned in the figure legends and table legends, and in addition in the main text (only lifespan experiment and tumour incidence)

(For large datasets, or papers with a very large number of statistical tests, you may upload a single table file with tests, Ns, etc., with reference to sections in the manuscript.)

**Group allocation**

- Indicate how samples were allocated into experimental groups (in the case of clinical studies, please specify allocation to treatment method); if randomization was used, please also state if restricted randomization was applied
- Indicate if masking was used during group allocation, data collection and/or data analysis

Please outline where this information can be found within the submission (e.g., sections or figure legends), or explain why this information doesn’t apply to your submission:

For the all experiments age-matched mice with different genotypes were compared, as described in the Material and methods section and figure legends.

**Additional data files (“source data”)**

- We encourage you to upload relevant additional data files, such as numerical data that are represented as a graph in a figure, or as a summary table
- Where provided, these should be in the most useful format, and they can be uploaded as “Source data” files linked to a main figure or table
- Include model definition files including the full list of parameters used
- Include code used for data analysis (e.g., R, MatLab)
- Avoid stating that data files are “available upon request”

Please indicate the figures or tables for which source data files have been provided:

None
